# High-Frequency Color Doppler Ultrasonographic Demonstration of Branch-Level Vascular Preservation and Peri-Graft Fluid After One-Sided Dorsal Onlay Urethroplasty

**DOI:** 10.3390/diagnostics16081199

**Published:** 2026-04-17

**Authors:** Daisuke Watanabe, Kazuki Yanagida, Masayuki Shinchi, Akio Mizushima, Akio Horiguchi

**Affiliations:** 1 Department of Urology, Koto Hospital, Tokyo 136-0072, Japan; k.yanagida.sr@juntendo.ac.jp; 2 Department of Molecular and Cellular Therapeutics, Juntendo University Graduate School of Medicine, Tokyo 113-8421, Japan; akiom@juntendo.ac.jp; 3 Division of Reconstruction Center for Trauma, Burn and Tactical Medicine, National Defense Medical College Hospital, Saitama 359-8513, Japan; impreza@ndmc.ac.jp; 4 Department of Urology, National Defense Medical College Hospital, Saitama 359-8513, Japan; shinchimasayuki@yahoo.co.jp

**Keywords:** urethroplasty, Kulkarni technique, one-sided dorsal onlay, penile ultrasonography, color Doppler imaging, vascular preservation, peri-graft fluid

## Abstract

One-sided dorsal onlay urethroplasty (Kulkarni technique) aims to preserve urethral vascularity by limiting urethral mobilization to a single side of the corpus spongiosum while maintaining contralateral vascular attachments. Although the theoretical advantage of vascular preservation is widely accepted, direct postoperative visualization of perfusion at the branch level has rarely been demonstrated using non-invasive imaging. We present a single representative case of an 82-year-old male with a 54 mm anterior urethral stricture who underwent one-sided dorsal onlay urethroplasty. Representative postoperative images illustrating the vascular-preserving principle of this technique and its ultrasonographic assessment are shown. Postoperative evaluation was performed on postoperative day 4 using a high-frequency small-footprint linear probe (Hitachi L53K, ARIETTA ultrasound system), enabling high-resolution superficial imaging of the penile shaft. Color Doppler ultrasonography demonstrated preserved perfusion on the non-dissected side, including identifiable cavernous urethral shunt flow and distinct urethral and spongiosal arterial branches within the corpus spongiosum. In contrast, no detectable Doppler signals were observed on the dissected side, which may reflect reduced perfusion following surgical mobilization. In addition, peri-graft fluid collections were visualized as hypoechoic regions adjacent to the graft bed, which may represent expected early postoperative findings. These findings highlight the feasibility of bedside branch-level vascular and peri-graft assessment using high-frequency ultrasonography, suggesting its potential utility for early postoperative clinical interpretation after one-sided urethral reconstruction.

**Figure 1:**
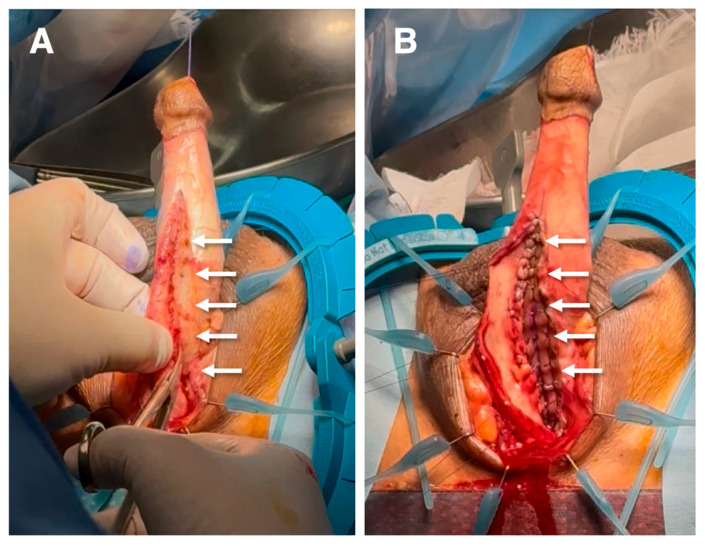
**Intraoperative demonstration of one-sided urethral mobilization and graft placement in one-sided dorsal onlay urethroplasty.** (**A**) Intraoperative view showing urethral mobilization and dissection limited to a single side of the corpus spongiosum. The contralateral attachments remain intact, preserving vascular connections to the urethral plate and surrounding spongiosal tissue. Arrows indicate the dissected urethral margin and preserved contralateral side. (**B**) Placement and suturing of the graft onto the corpus cavernosa. The graft is secured to the tunica of the corpus spongiosum without circumferential urethral mobilization. This approach maintains unilateral vascular integrity while expanding the urethral lumen. The preserved side is not mobilized, thereby minimizing disruption of spongiosal blood supply. These intraoperative images illustrate the fundamental principle of the one-sided dorsal onlay (Kulkarni) technique: unilateral dissection with contralateral vascular preservation [1,2].

**Figure 2:**
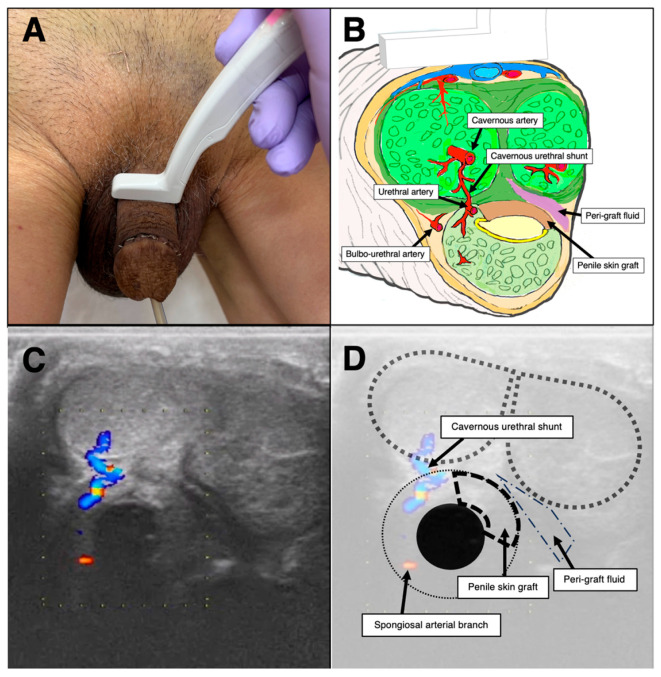
**Ultrasonographic visualization of vascular preservation and peri-graft assessment after one-sided dorsal onlay urethroplasty:** (**A**) Bedside ultrasonographic examination of the penile shaft using a high-frequency small-footprint linear probe (Hitachi L53K, ARIETTA ultrasound system, Tokyo, Japan). The probe’s compact footprint enables optimal contact with the curved penile surface and facilitates high-resolution imaging of superficial penile structures. (**B**) Schematic transverse anatomy of the penis demonstrating the corpora cavernosa, corpus spongiosum, urethral lumen, and detailed vascular anatomy. The cavernous arteries give rise to helicine branches within the corpora cavernosa. The urethral artery and its spongiosal arterial branches supply the corpus spongiosum. Communicating branches between the corpora cavernosa and the corpus spongiosum (cavernous urethral shunts) are illustrated, reflecting vascular interconnections between erectile tissues. In the one-sided dorsal onlay technique, urethral mobilization is limited to the left side (dissected side), whereas the contralateral (right) side remains undissected, preserving vascular attachments, as illustrated by the color-coded schematic. (**C**) Representative transverse postoperative color Doppler ultrasonographic image. Doppler assessment is qualitative, based on the presence or absence of detectable flow signals at the branch level. Distinct intraparenchymal arterial signals are observed on the non-dissected side within the corpus spongiosum and adjacent cavernosal tissue. In contrast, no detectable Doppler flow signal is observed on the dissected side, suggestive of relatively reduced perfusion following surgical mobilization. (**D**) Annotated sonographic image correlating anatomical structures. Dotted outlines indicate the corpora cavernosa; the dashed semicircle highlights the corpus spongiosum surrounding the urethral catheter. Color Doppler signals are observed on the non-dissected side, including flow within the cavernous urethral shunt and spongiosal arterial branches. The dissected side shows absence of detectable Doppler signals. Hypoechoic regions adjacent to the graft bed are noted on the dissected side, which may represent expected early postoperative peri-graft fluid rather than pathological findings, consistent with the patient’s uneventful clinical course.

## Data Availability

The datasets used and analyzed in this paper are available from the corresponding author upon reasonable request.
